# Recent Advances in Ureteral Tissue Engineering

**DOI:** 10.1007/s11934-014-0465-7

**Published:** 2014-11-11

**Authors:** Paul K. J. D. de Jonge, Vasileios Simaioforidis, Paul J. Geutjes, Egbert Oosterwijk, Wout F. J. Feitz

**Affiliations:** Department of Urology, Radboud Institute for Molecular Life Sciences, Radboud University Medical Center, P.O. Box 9101, Geert Grooteplein 26/28, 6525 GA Nijmegen, The Netherlands

**Keywords:** Ureter, Regeneration, Reconstruction, Tissue engineering, Regenerative medicine, Stem cells, Cells, Pre-implantation, Biomaterials, Animals

## Abstract

Reconstruction of long ureteral defects often warrants the use of graft tissue and extensive surgical procedures to maintain the safe transport of urine from the kidneys to the urinary bladder. Complication risks, graft failure-related morbidity, and the lack of suitable tissue are major concerns. Tissue engineering might offer an alternative treatment approach in these cases, but ureteral tissue engineering is still an underreported topic in current literature. In this review, the most recent published data regarding ureteral tissue engineering are presented and evaluated, with a focus on cell sources, implantation strategies, and (bio)materials.

## Introduction

Advances in endourology led to an increase in the number of ureteroscopies and nephroscopies during the last two decades worldwide. The complications of these procedures are often underreported, possibly due to lack of early recognition or short-term postoperative follow-up [1–3]. Strictures after ureteroscopy occur with an estimated frequency up to 3.5 %, which is more often than avulsion or major perforation [4, 5•, 6]. In around 1 % of the gynecological procedures, a ureteral injury occurs, which is estimated to account for up to 73 % of all ureteral injuries [3, 7, 8]. In addition to these iatrogenic injuries, trauma can result in ureteral damage. A large retrospective analysis in the USA showed that 2.6 % of all urogenital traumas involved the ureter between 2002 and 2006 [9]. The specific anatomic characteristics of the ureter, such as the segmental vascular supply, can be easily damaged, and the lack of native tissue limits surgical ureteral reconstruction. To repair the long ureteral defects, where an end-to-end anastomosis is not feasible for the urologist, several techniques have been introduced such as a ureteroneocystostomy, a Boari flap, ileal interposition, and renal autotransplantation. Ultimately, when surgical expertise is not available or the aforementioned techniques do not succeed, an undesirable nephrectomy is the only option [10, 11].

Tissue engineering might offer new treatment approaches in ureteral reconstruction to optimize the outcome in complicated cases, which currently have complication rates up to 25 % [12]. The number of published manuscripts dealing with tissue engineering applications of the urinary system is quite extensive, particularly for urinary bladder reconstruction, but the number of research groups that focus on tissue engineering of the ureter is limited, which suggests that the development of an artificial construct suitable for ureteral reconstruction is challenging. Additionally, there might be less incentive to investigate tissue engineering approaches as the incidence of long ureteral injuries is lower than urethra or bladder injuries.

## Ureteral Tissue Engineering

In a recent review on ureteral tissue engineering [13], it was demonstrated that the evidence in the literature was inconclusive about the optimal tissue engineering approach to treat long ureteral injuries. Furthermore, compared to other parts of the urogenital tract, very few tissue engineering studies were performed. Most studies focused on tubular(ized) small intestinal submucosa (SIS) without cellular pre-seeding [14–20]. Other materials included collagen [21, 22] and Gore-Tex [23, 24]. In general, collagen and SIS, but not Gore-Tex, were capable of facilitating some degree of urothelium and smooth muscle regeneration. However, fibrosis occurred in most cases.

In this review, we present recent developments in ureteral tissue engineering and discuss currently used materials, construct design, cell sources, and implantation techniques. In addition to the recent literature reviews on ureteral tissue engineering, a Medline search was performed for papers published in the last 3 years using a previously published tissue engineering filter [25] combined with the MeSH term ureter. An overview of the recent studies is presented in Table 1 in which we focused on the early post-implantation complications and the presented solutions.

**Table 1:** Recent ureteral tissue engineering studies

Authors	Animal model	Biomaterial	Cell seeded	Length (cm)	Technique	Outcome
Xu et al. [26]	Rats (M)	PLLA	No	0.9	S, T	I_1_, V
Shi et al. [27]	Mice (F)	PLLA, Collagen	hADSC	–	S, T	hUC
Fu et al. [28]	Mice (M)	PLLA, Collagen	hUC	1.0–1.5	S, T	hUC
Zhang et al. [29]	Dogs (F)	Autologous graft	No	3.0	P, T	UC, SMC, V
Salehipour et al. [30]	Dogs (M)	AM	No	3.0	T*	L, H, F, I_2_
Zhao et al. [31••]	Rabbits (F)	VECM	ADSC	3.0	T	UC, SMC
Liao et al. [32••]	Rabbits (M)	BAM	MSC, SMC	4.0	P, T*	I_1_, UC, SMC
De Jonge and Simaioforidis et al. (unpublished)	Pigs (F)	Collagen	UC, SMC	5.0	T	UC, SMC, L, F, H

*PLLA* poly(l-lactic acid), *AM* amniotic membrane, *VECM* vessel extracellular matrix, *BAM* bladder acellular matrix, *(h)ADSC* (human) adipose derived stem cell, *MSC* mesenchymal stem cell, *(h)UC* (human) urothelial cell, *SMC* smooth muscle cell, *S* subcutaneous implantation, *P* pre-implantation, *T* tubular, T* tubularized, *I* inflammation (*I*_*1*_ mild, *I*_*2*_ severe), *V* vascularization, *F* fibrosis, *H* hydronephrosis, *L* urine leakage

## Ureteral Defect Repair

To study the effect of a tissue-engineered construct on the regeneration of the ureter, it is imperative to test the constructs in a ureteral defect model. Nevertheless, in three recent studies, the authors refrained from implantation of tissue-engineered constructs in an induced ureteral defect model. Instead, the authors performed subcutaneous implantations in rats (Xu, et al. [26]) and mice (Shi, et al. [27] and Fu, et al. [28]). While these studies showed the potential of pre-implantation for ureteral replacement, information about the behavior of the construct as ureteral replacement is lacking. In the intracorporeal environment, constructs are exposed to the toxic effects of urine and various mechanical forces [5•]. All three studies used a similar spiral poly(**l**-lactic acid) (PLLA) stent as the backbone of their construct. Xu et al. [26] implanted the spiral PLLA stents in the subcutis and used the body as a natural bioreactor to generate a tissue fleece around the stent. The newly formed tissue was then decellularized and re-seeded with primary urinary bladder urothelial cells. Cell proliferation was similar compared to SIS. Shi et al. [27] and Fu et al. [28] combined the same PLLA stent with electrospun collagen to improve cell attachment and cell proliferation. Before subcutaneous implantation in athymic mice, the final constructs were seeded with human adipose-derived stem cells (hADSCs) (Shi*,* et al. [27]) or human urothelial cells (hUC) (Fu, et al. [28]). Both authors were able to detect viable human cells 2 weeks post-implantation, demonstrating that the cells could survive the procedure. These studies solely indicate that the subcutis might be a suitable pre-implantation site to generate a tubular pre-vascularized autologous tissue, which may prevent fibrosis when attempting to repair the ureter.

## Cell Sources

The use and necessity of cell seeding of tissue-engineered constructs has been a matter of debate, but once it is considered, many options exist. Embryonic stem cells are highly controversial due to their origin and the risk of tumor formation. A safer and less controversial option is the use of autologous cells when available. Tissue biopsies can yield differentiated primary cells or multipotent cells like mesenchymal or adipose-derived stem cells. Most early studies in ureteral tissue engineering used bare scaffolds, and almost all of them resulted in fibrosis, which may indicate the necessity of cell seeding [14–19, 21–24, 33]. This is supported by previous statements that cell seeding is required for large defects (>1.0 cm from the wound edge) to promote tissue regeneration and to prevent scar formation [34]. In most recent studies, cell seeding or pre-implantation of the scaffolds was explored to improve regeneration [27–29, 31••, 32••].

Fu et al. [28] used primary urothelial cells, isolated from patients that underwent nephrectomy, which were seeded on spiral PLLA stents and subcutaneously implanted in a nude mouse model for 2 weeks. The grafts resulted in a thin tissue capsule in which the seeded cells were still present and viable. This successful approach is relatively straightforward, albeit time consuming since it takes 4 weeks before ureter reconstruction can be performed; the cells are expanded for 2 weeks, followed by pre-implantation for 2 weeks.

**Fig. 1:**
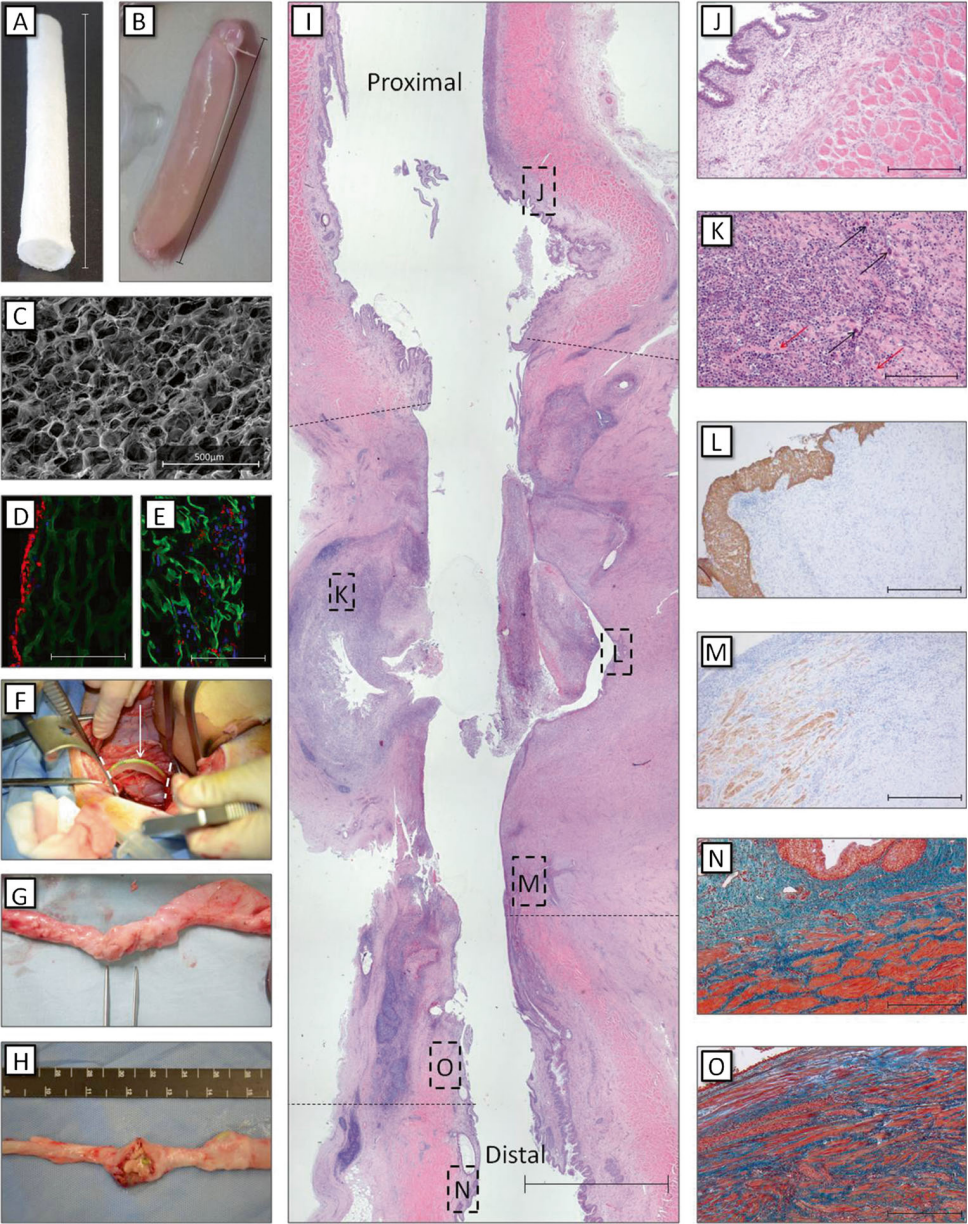
Implantation strategy and outcome after ureteral reconstruction using tubular collagen scaffolds. **a**, **b** Macroscopic overview of a tubular 0.5 % type-I collagen scaffold (*length* = 6 cm, Ø = 6 mm). **c** SEM overview of the scaffold surface, which was highly porous, facilitating cell penetration into the scaffold (*scale bar* = 500 μm) **d** Immunofluorescent staining for collagen (*green*), nuclei (*blue*), and RCK103 (*red*) of a cell-seeded scaffold. Urothelial cells (RCK103 positive) were lining the scaffold (*scale bar* = 400 μm). **e** Immunofluorescent staining for collagen (*green*), nuclei (*blue*), and α smooth muscle actin (*red*) of a cell-seeded scaffold. Smooth muscle cells (α smooth muscle actin positive) were found throughout the scaffold (*scale bar* = 400 μm). **f** The scaffolds were implanted by end-to-end anastomosis. **g**, **h** Ureteral regeneration was evaluated after 1 month. Four animals showed intact ureters (**g**), while seven animals presented with defects or dissections (**h**). **i** Histological overview of a regenerating ureter (*scale bar* = 5 mm). *Black dotted lines* indicate the anastomosis sites. Specific locations are highlighted (*J*–*O)*. **j** Hematoxylin and eosin (HE) staining of the native ureter (*scale bar* = 400 μm). **k** Inflammatory response in the regenerating tissue near scaffold remnants (*red arrows*). Mostly lymphocytes, a few granulocytes, and some multinucleated giant cells (*black arrows*) were observed (*scale bar* = 200 μm). **l** Pancytokeratin staining in the middle of the neo-ureter, indicating the presence of (multilayered) urothelium (*scale bar* = 400 μm). **m** Smoothelin staining near the anastomosis site, indicating ingrowth and maturation of smooth muscle tissue into the neo-ureter (*scale bar* = 400 μm). **n** Masson’s trichrome staining of the native ureter (*scale bar* = 400 μm). **o** Masson’s trichrome staining near the anastomosis site, indicating the ingrowth of new muscle tissue (*scale bar* = 400 μm)

A faster approach would be the implantation of only the cell-seeded construct. To investigate this approach, we implanted 5.0-cm-long highly porous tubular 0.5 % type-I collagen constructs to repair a full ureteral defect in 11 female Landrace pigs (unpublished data). In brief, primary urothelial (UC) and smooth muscle cells (SMCs) were isolated from porcine urinary bladder biopsies [35]. First, the scaffolds were homogenously seeded with urinary bladder-derived SMCs, followed by luminal seeding of urinary bladder-derived UC. The right ureter was approached and mobilized through a midline incision, and a 5.0-cm segment of the ureter was removed and replaced with an equally sized scaffold. A 6 Fr double-J stent was placed to facilitate urinary flow, and animals were followed up to 4 weeks. In 7/11 pigs, abdominal swelling due to urine leakage was observed after 2–3 weeks. The other animals developed strictures and hydronephrosis despite the presence of the stent. Upon analysis, it became clear that the urine leakage could be attributed to insufficient mechanical strength of the collagen scaffolds, which resulted in ruptures or dissections of the scaffolds. In the animals where the scaffold remained patent, the scaffold was mostly covered by a single layer of urothelial cells. Extensive neovascularization and some SMC ingrowth were observed (Fig. 1). Although the collagen construct with primary urinary bladder cells was suitable for ureteral reconstruction, we can conclude that back-bone biodegradable synthetic materials are needed to bear mechanical loads when attempting to repair an unsupported, mobile organ like the ureter.

One of the major disadvantages of primary cells, especially for urothelium, is that the cells cannot safely be harvested in case of possible malignancies [36]. Also, suitable tissue may not always be available for cell isolation. Therefore, alternative cell sources are being explored with a focus on mesenchymal (MSCs) and adipose-derived stem cells (ADSCs). These cells can differentiate into multiple cell lineages, including muscle and epithelium, without the risk of tumor formation [37–40]. Additionally, they are associated with anti-inflammatory properties and the capability to produce several cytokines that are associated with normal wound healing [41].

Zhao et al. [31••] isolated ADSC from rabbits and differentiated these towards a SMC phenotype before cell seeding and implantation. The cells were seeded on decellularized rabbit abdominal aorta to prepare a vascular extracellular matrix (VECM). Cell-seeded scaffolds were used to replace a 3.0-cm-long defect of the rabbit ureter. After 16 weeks, the defect was characterized by a well-organized muscle layer and stratified urothelium similar to native tissue. Strictures and hydronephrosis were absent. The authors attributed the positive results to the stimulating effect of ADSC on SMC proliferation and differentiation and the use of a graft containing many natural occurring growth factors [42]. Additionally, Shi et al. [27] showed that human ADSC can survive and maintain their phenotype for at least 2 weeks when implanted subcutaneously in nude mice, showing the possibility to use these cells for their stimulating properties in time. Alternatively, MSC can be used. These cells possess similar properties as ADSC but are isolated from the bone marrow. The harvesting procedure of these cells is painful, the differentiation potential decreases with age, and the number isolated cells is limited compared to ADSC [43, 44]. Liao et al. [32••] used MSC in combination with SMC seeded on bladder acellular matrix (BAM) to repair a 4.0-cm ureteral defect in rabbits. The BAM was seeded with bone marrow-derived mesenchymal stem cells (MSCs) on one side and urinary bladder-derived SMCs on the other side to create tissue-engineered tubular grafts (TETGs). The TETG was tubularized around a catheter and pre-implanted in the omentum of rabbits for 2 weeks. During this pre-implantation, the MSC differentiated and formed a single-cell-layered epithelium. Next, the ureteral defect was repaired, where a multilayer urothelium with central neovascularization was observed after 16 weeks. No strictures or hydronephrosis was observed, even though the ureteral catheter was removed 6 weeks postoperatively. Without MSC, the ureter repair resulted in scar formation and severe hydronephrosis. The investigators reasoned that the formation of the single-layered epithelium during the pre-implantation phase might have protected the surrounding tissue against urine.

These examples show the potential of stem cells as an alternative cell source for ureteral tissue engineering when insufficient donor tissue is available.

## Full-Circumference Ureteral Defect Repair

Major ureteral reconstructions are required when a complete segment of the ureter needs to be replaced. Onlay graft repair is most often impossible and can only be applied in stricture repair. It is therefore not surprising that the majority of the studies focus on the repair of long (relative to the total length of the ureter) defects using tubular or tubularized constructs. Zhang et al. [29] used 3.0-cm-long tubular autologous connective tissue that was formed after the implantation of silicon tubes in the peritoneal cavity of dogs. By maintaining one third of the native ureter, the investigators kept adequate vascularization and they managed to generate new tissue which was similar to the normal ureter. After 12 weeks, the tubular construct was completely lined by multilayered urothelium and presented with an organized muscle layer and mucosal folds. While these results are very promising, one has to realize that it is unlikely that one third of healthy ureter is present in the clinical situation, e.g., in case of severe adhesions or prolonged avascularity. It is generally accepted that tissue ingrowth after 1.0 cm becomes increasingly difficult and is likely to be accompanied by fibrosis [34]. By maintaining the ureteral segment, the authors avoided this challenge.

## Pre-implantation

The lack of functional urothelium and adequate vascularization may contribute to stricture formation and fibrosis as urine can freely damage the regenerating tissue [5•, 45]. Pre-implantation promotes vascularization and helps to maintain the viability of the seeded cells in vivo. Different pre-implantation sites have been used for various applications, including the omentum [46, 47], peritoneum [48, 49], and subcutis [49, 50]. Zhang et al. [29] and Liao et al. [32••] took advantage of pre-implantation before repairing a ureteral defect. Zhang et al. exploited pre-implantation to create a tubular scaffold from the fibrous capsule which was formed in the peritoneal cavity. Liao et al. [32••] used omental pre-implantation as an in vivo bioreactor to increase neovascularization in the construct. Additionally, it allowed the formation of a one-layer epithelial structure which may protect the construct after implantation in the toxic urine-rich environment.

Ideally, when harvesting the pre-implanted material, the newly formed blood vessels should remain intact. A mobile pre-implantation site close to the ureteral defect repair site, like the greater omentum, might be suitable for this as flaps can easily be mobilized most of the time. Although pre-implantation techniques are promising, they are time consuming and require a second surgical procedure. Therefore, they may not always be applicable in case of acute problems and unplanned procedures, which is often the case with ureteral trauma.

## Decellularized Tissue and Synthetic Polymers

A variety of materials has been used as scaffolding material. Most studies used decellularized tissues as opposed to “man-made” scaffolds in the past decades. The advantages of decellularized tissues include preservation of the native tissue architecture and inclusion of tissue-specific growth factors and other signaling molecules [42]. In the past, SIS has been the decellularized tissue of choice. The results, however, were not optimal in ureteral tissue engineering. Recently, Salehipour et al. [30] used amniotic membrane (AM), which is known for its anti-inflammatory properties, as a biomaterial to reconstruct long ureteral defects. In dogs, a 3.0-cm segment of the ureter was replaced by tubularized decellularized AM. Two out of seven animals died due to urine leakage, and another animal showed severe hydronephrosis, acute and chronic inflammation, and the formation of granulation tissue. The other animals presented with mild pelvicaliectasis and fibrosis of the reconstructed segment with lymphatic and granulocytic infiltration. Where Koziak et al. [51] showed encouraging results when AM was used as an onlay graft in 2007, the authors of this study concluded that AM did not act as a favorable material when used in full defects. This result was similar to a previous study by Osman et al. in 2004 [33]. Decellularized blood vessels and bladder acellular matrix have recently been used with promising results, but these results may also be caused by the use of stem cells and pre-implantation techniques, something that was not done in combination with SIS for the ureter [31••, 32••].

Besides decellularized tissues, man-made scaffolds can be used. The advantage of these scaffolds is a higher degree of plasticity, good mechanical properties, and they are well defined. Most materials can be prepared in any shape (e.g., flat, film, or tube) or size, and different proteins and bioactive molecules can be added as demonstrated in the recent publications using spiral PLLA stents in combination with collagen and our tubular collagen scaffolds. When improvements such as increased mechanical strength are required, these man-made scaffolds can easily be tailored compared to decellularized tissue.

## Animal Models

In recent ureteral replacement animal studies, rabbits [31••, 32••], dogs [29, 30], and in our case, pigs (unpublished) were used, while subcutaneous implantation studies were performed in rats [26] or mice [27, 28]. The pig is the preferred model because the abdominal anatomy of pigs and humans is similar [52, 53]. Nevertheless, the lack of recent pig studies might be associated with the high incidence of fibrosis and fast growth of the animal, as mostly fast-growing young pigs are used. This may influence tissue regeneration and cause mechanical stress on the tissue constructs. The ideal animal should have a similar size and abdominal anatomy as humans, be fully grown, and have similar wound healing characteristics. Potential candidate animals include goats, sheep, cattle, and horses. In general, randomized controlled trials preceded by extensive toxicity studies are required before a new technique is widely used in the clinic. However, in tissue engineering, it is unethical to perform safety studies in healthy patients and there is often a lack of golden standard treatments. Therefore, choosing the right animal models is critical to predict the expected clinical outcome as good as possible [54].

## Conclusions

Ureteral reconstruction should focus on the maintenance of safe urine transport from the kidney to the bladder. Fast development of a vascular system, a functional smooth musculature, and a urothelial barrier is critical for the success of constructs as the lack of these layers may result in strictures and hydronephrosis, even when stents are used. In the past few years, clear advancements have been made in ureteral tissue engineering. Specifically, the cell source, implantation techniques, and new biomaterials have improved the tissue engineering of the ureter. Decellularized tissues or scaffolds with added natural proteins and other molecules may perform better than simple scaffolds; however, these were only studied in the context of stem cells. Despite these advancements, published research in the area of ureteral tissue engineering is scarce. Many recent studies do not address the behavior of the constructs in a ureteral replacement setting. To increase our knowledge on the effect of different materials, cell sources, and implantation techniques, future studies should attempt to repair a full ureteral defect. Current literature suggests that the use of mesenchymal and adipose-derived stem cells, seeded on any type of mechanically suitable bioactive material, is optimal for ureteral regeneration. In addition, pre-implantation of these constructs in the omentum may improve the final outcome by increasing vascularization and triggering stem cell differentiation. However, when using the body as an in vivo bioreactor, the long incubation time may be problematic in ureteral repair. Finally, different pre-clinical animal models should be evaluated to prevent species-related result bias prior to commencing clinical trials.
